# Crystal structure and SUMO binding of Slx1-Slx4 complex

**DOI:** 10.1038/srep19331

**Published:** 2016-01-20

**Authors:** Fu-Ming Lian, Si Xie, Chengmin Qian

**Affiliations:** 1 School of Biomedical Sciences, The University of Hong Kong, Hong Kong, China

## Abstract

The SLX1-SLX4 complex is a structure-specific endonuclease that cleaves branched DNA structures and plays significant roles in DNA recombination and repair in eukaryotic cells. The heterodimeric interaction between SLX1 and SLX4 is essential for the endonuclease activity of SLX1. Here, we present the crystal structure of Slx1 C-terminal zinc finger domain in complex with the C-terminal helix-turn-helix domain of Slx4 from *Schizosaccharomyces pombe* at 2.0 Å resolution. The structure reveals a conserved binding mechanism underling the Slx1-Slx4 interaction. Structural and sequence analyses indicate Slx1 C-terminal domain is actually an atypical C4HC3-type RING finger which normally possesses E3 ubiquitin ligase activity, but here is absolutely required for Slx1 interaction with Slx4. Furthermore, we found the C-terminal tail of *S. pombe* Slx1 contains a SUMO-interacting motif and can recognize Pmt3 (*S. pombe* SUMO), suggesting that Slx1-Slx4 complex could be recruited by SUMOylated protein targets to take part in replication associated DNA repair processes.

Multi-domain containing scaffold protein SLX4 (also known as BTBD12) has been shown to interact with a group of proteins including SLX1 (also known as GIYD2), MUS81-EME1 and XPF-ERCC1 and coordinates these endonucleases for the resolution of Holiday junctions or interstrand crosslinks structures^1^,^2^,^3^,^4^,^5^,^6^,^7^,^8^. Additionally, SLX4 has also been reported to bind to telomeric protein TRF2 to preserve telomere^9^. HIV-1 protein Vpr associates with SLX4 directly and activates SLX4 complex by VPRBP-DDB1-CUL4 E3 ubiquitin ligase complex, resulting in G2/M arrest and allow HIV to avoid innate immune sensing^10^. Furthermore, SLX4 contains tandem UBZ4 domains and multiple SUMO-interacting motifs (SIMs) that have been demonstrated to be critical for SLX4 to bind to specific ubiquitinated or SUMOylated genome integrity maintenance proteins to process different types of DNA lesions^11^,^12^,^13^,^14^. Therefore, SLX4 functions as a toolkit to regulate several DNA damage response pathways and maintain genome stability. Interestingly, mutations in SLX4 have recently been identified in patients with a new subtype of Fanconi anemia (Fanconi anemia-P), hence SLX4 has been renamed as *FANCP*^11^,^15^. Although considerable progress has been made in understanding how SLX4-coordinated protein complex functions to preserve genome integrity, the precise mechanism by which SLX4 complex resolves the Holliday junctions at the molecular level has yet to emerge.

SLX4 and SLX1 were originally identified in the screen for synthetic lethality with the defective RecQ helicase Sgs1 (BLM in human) in yeast^16^. Later on yeast Slx1-Slx4 complex was found to cleave branched DNA structures such as Y-forks, 3′ flaps, 5′ flaps, and Holliday junction structures, but with the preference to 5′ flaps^17^. A number of observations have suggested that yeast Slx1-Slx4 complex plays a role in maintaining the integrity of the ribosomal DNA loci during S phase^17^,^18^,^19^. Slx1 harbors an N-terminal catalytic GIY-YIG endonuclease domain followed by a C-terminal zinc finger motif (Supplementary Fig. S1A). Individual Slx1 has very weak nuclease activity, while association with Slx4 could dramatically enhance its enzymatic activity^1^,^17^. Slx1 C-terminal zinc finger and Slx4 C-terminal helix-turn-helix domain are well conserved throughout evolution (Supplementary Figs S1A and B), and have been mapped out to be the minimal fragment of each protein required for the complex formation^1^,^2^. More recent studies have demonstrated that both human and yeast Slx1-Slx4 possess robust Holliday junction resolvase activity^1^,^2^,^3^,^20^.

Here, we report the crystal structure of the *Schizosaccharomyces pombe* Slx1 C-terminal RING finger domain in complex with Slx4 C-terminal four-helix bundle domain, which provides structural insights into the Slx1-Slx4 complex formation. In addition, we uncover the C-terminal tail SUMO-interacting motif of *S. pombe* Slx1 binds SUMO, which imply that *S. pombe* Slx1-Slx4 complex can be recruited by SUMOylated proteins to involve in the DNA damage response.

## Results

### Overall structure of Slx1^RING^-Slx4^CCD^ complex

**Figure 1:**
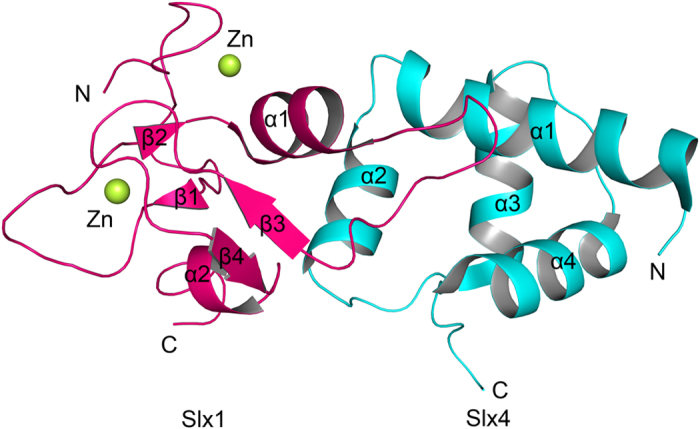
Cartoon representation of Slx1^RING^-Slx4^CCD^ complex structure. Slx1^RING^ and Slx4^CCD^ are colored in pink and cyan, respectively. Green spheres indicate zinc ions. Loop1 and helix α1, two U-shaped loops that connect antiparallel β1-β2 and β3-β4 sheets contribute to the coordination of two zinc ions.

We purified the complex of Slx1 C-terminal zinc finger (Slx1^RING^, residues: 176–247) with Slx4 C-terminal conserved domain (Slx4^CCD^, residues: 352–419) co-expressed in *Escherichia coli*. The Slx1^RING^-Slx4^CCD^ complex exists as a heterodimer in solution indicated by analytical size-exclusion chromatography. To provide structural insights into the protein component and complex architectures, we determined the crystal structure of Slx1^RING^-Slx4^CCD^ complex to 2.0 Å resolution. Each asymmetric unit of the crystal is composed of two nearly identical Slx1-Slx4 heterodimers, with residues Val178–Thr246 of Slx1 and Ile352-Ser417 of Slx4 well fitted in the final structure model (Fig. 1). Slx1^RING^ adopts a compact α/β structural fold, consisting of two two-stranded antiparallel β1-β2 and β3-β4 sheets and two α helices. Slx1^RING^ possesses a RING finger structural topology, which includes the pattern of chelating two zinc atoms and has a characteristic helix α1. This conserved helix is involved in E2 recognition in RING domains with E3 ubiquitin ligase activities. Homology search with Dali server^21^ indicated that Slx1 zinc finger shares the most similar fold with a consistent series of RING finger proteins (e.g. FANCL, RNF4, and RING1b)^22^,^23^,^24^, while Slx1 RING finger contains an unusual long loop following helix α1 which is directly involved in the interaction with Slx4^CCD^. Slx4^CCD^ is folded as a helix-turn-helix domain containing a four-helix bundle (α1–α4), with residues from helices α1, α2, and α4 constituting the Slx1^RING^ binding interface.

### Insights into Slx1^RING^-Slx4^CCD^ complex interface

**Figure 2:**
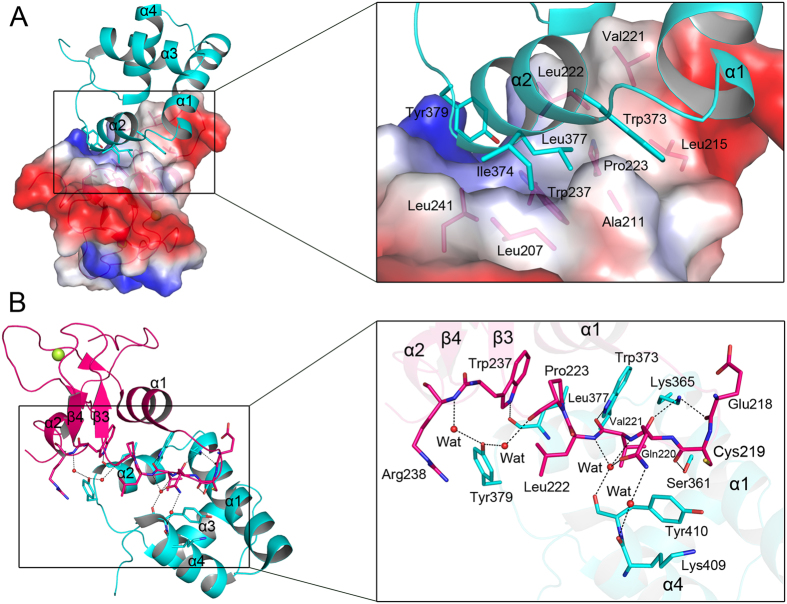
The Slx1^RING^-Slx4^CCD^ complex interface. **(A)** Detailed representation of complex interface involved in hydrophobic interactions. Slx4^CCD^ is shown as cartoon diagram colored in cyan, and Slx1^RING^ is indicated as cartoon diagram colored in pink and translucent electrostatic surface potential representations. The involved residues are shown as sticks. (**B)** Detailed representation of complex interface involved in hydrogen bonds. Slx1^RING^ and Slx4^CCD^ are colored in pink and cyan, respectively. The involved residues are shown as sticks. Water molecules are shown as red balls.

Slx1^RING^-Slx4^CCD^ heterodimer buries a total accessible surface area of ~1470 Å^2^ (Fig. 2). Both hydrophobic interactions and hydrogen bonding contribute to the complex formation. Indeed, both Slx4^CCD^ and Slx1^RING^ have the hydrophobic patch to constitute the majority part of binding interface (Fig. 2A). These residues constituting hydrophobic patches are conserved along the evolution in both proteins as revealed by multiple-sequence alignment (Supplementary Figs S1A and B). Residues Trp373, Ile374, and Leu377 in Slx4^CCD^ helix α2 and Tyr379 located at the subsequent loop form the majority of the hydrophobic patch on Slx4 C-terminal helix-turn-helix domain. The Slx1^RING^ residues from helix α1 (Leu207, Ala211, and Leu215), the loops after helix α1 but before helix α2 (Val221, Leu222, Pro223, and Trp237), and helix α2 (Leu241) contribute to the majority of the hydrophobic patches. The interface is further stabilized by several hydrogen bonds between the residues from Slx1^RING^ and Slx4^CCD^. (Fig. 2B). Main-chain carbonyl groups of Glu218, Cys219, Gln220, and Val221 of Slx1^RING^ make hydrogen-bonding with side chains of Lys365, Ser361, and Trp373 of Slx4^CCD^ respectively. A hydrogen bond is formed between the side-chain indole nitrogen of Trp237 of Slx1^RING^ and the main-chain carbonyl group of Leu377 of Slx4^CCD^. In addition, the side-chain hydroxyl group of Slx4^CCD^ Tyr379 forms water-mediated hydrogen bonds with Slx1^RING^ backbone amino group of Arg238 and carbonyl group of Pro223. Main-chain carbonyl groups of Slx4^CCD^ Lys409 and Tyr410 contribute to water-mediated hydrogen-bonding with Slx1^RING^ side-chain of Gln220 and main-chain amino groups of Val221 and Leu222.

Structural comparison of our Slx1^RING^-Slx4^CCD^ complex with the recently determined *Candida glabrata* Slx1-Slx4^CCD^ structure revealed that two structures share a similar Slx1^RING^ or Slx4^CCD^ structural fold with overall RMSD of 2.0 and 1.7 Å over 67 and 64 Cα atoms (Supplementary Fig. S2)^20^. However, it is worthy to note that the long loop right after helix α1 is broken probably due to the high flexibility in *Candida glabrata* Slx1-Slx4^CCD^ structure, but is well defined in *S. pombe* Slx1^RING^-Slx4^CCD^ structure. The long loop (Thr216-Ile224) with negative electrostatic potential protrudes and binds to Slx4^CCD^ helix α1 area with strong positive electrostatic potential (Fig. 2A), which contributes to the formation of complex interface (Fig. 2).

### The C-terminal tail SUMO-interacting motif of *S. pombe* Slx1 recognizes SUMO

**Figure 3 f3:**
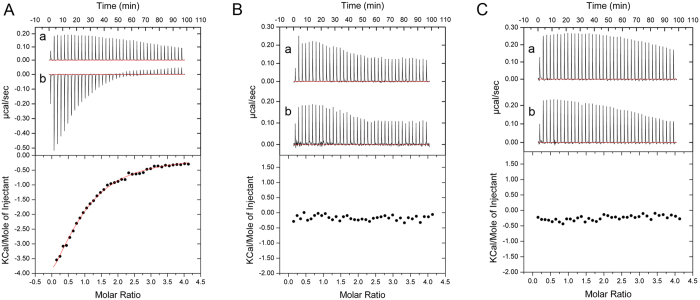
Representative ITC titrations of Pmt3 into Slx1^RING+CT^-Slx4^CCD^, Slx1^RING^-Slx4^CCD^, and Slx1^RING+CT SIMmut^-Slx4^CCD^. (**A)** The upper panel shows the experimental measurements of titrating Pmt3 into the buffer (a), and into Slx1^RING+CT^-Slx4^CCD^ (b). The lower panel shows integrated binding isotherms of calorimetric titrations and the fitted curve. (**B)** The upper panel indicates the experimental measurements of titrating Pmt3 into the buffer (a), and into Slx1^RING^-Slx4^CCD^ (b). The lower panel indicates the integrated isotherms of titrations. (**C)** The upper panel shows the experimental measurements of titrating Pmt3 into the buffer (a), and into Slx1^RING+CT SIMmut^-Slx4^CCD^ (b). The lower panel indicates the integrated isotherms of titrations.

**Figure 4:**
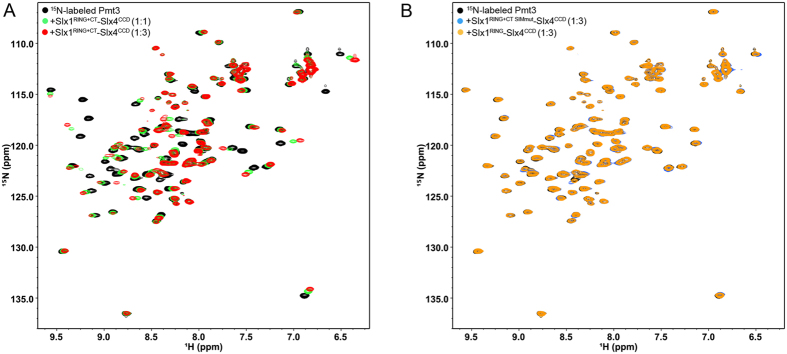
NMR titrations of Slx1^RING+CT^-Slx4^CCD^, Slx1^RING^-Slx4^CCD^, or Slx1^RING+CT SIMmut^-Slx4^CCD^ into ^15^N-labeled Pmt3. (**A)** Overlay of ^1^H-^15^N HSQC spectra of Pmt3 amide resonances in the free (black) and in the presence of Slx1^RING+CT^-Slx4^CCD^ with molar ratios 1:1 (green) and 1:3 (red). Chemical shift changes indicate the binding between Pmt3 and Slx1^RING+CT^-Slx4^CCD^. **(B)** Overlay of ^1^H-^15^N HSQC spectra of Pmt3 amide resonances in the free (black) and in the presence of Slx1^RING^-Slx4^CCD^ (orange) and Slx1^RING+CT SIMmut^-Slx4^CCD^ (blue) with molar ratios 1:3.

Human SLX4 contains tandem ubiquitin binding zinc fingers (UBZ) that bind to ubiquitin and are essential for [Fig f3]interstrand crosslink repair^11^. Interestingly, more recently several studies has indicated human Slx4 contains multiple SUMO-interacting motifs binds to SUMO to enhance the interaction with specific DNA repair factors to process replication-associated DNA damage^12^,^13^,^14^. Unexpectedly, we found a SUMO-interacting motif (-IIDLE-, residues 264-268) located at the C-terminal tail of *S. pombe* Slx1. To evaluate the SUMO binding possibility of Slx1, we employed isothermal titration calorimetry (ITC) to measure the dissociation constant of the C-terminal domain of Slx1 (Slx1^RING+CT^, residues: 176-271)-Slx4^CCD^ complex binding to Pmt3 (*S. pombe* SUMO). ITC titrations showed that Slx1^RING+CT^-Slx4^CCD^ binds to Pmt3 with a *K*_D_ of 78.3 ± 9.9 μM (Fig. 3A). However, neither Slx1^RING^ (C-terminal tail deleted, residues: 176-247)-Slx4^CCD^ nor Slx1^RING+CT SIMmut^ (IIDLE mutated to AADAE)-Slx4^CCD^ could bind to Pmt3 (Fig. 3B,C). Thus Slx1 C-terminal IIDLE motif is critical for the SUMO binding. This conclusion is further verified by nuclear magnetic resonance (NMR) titrations of Slx1^RING+CT^-Slx4^CCD^, Slx1^RING^-Slx4^CCD^, or Slx1^RING+CT SIMmut^-Slx4^CCD^ into ^15^N-labeled Pmt3 (Fig. 4). A number of residues showed significant chemical shift perturbations when Slx1^RING+CT^-Slx4^CCD^ was titrated into the ^15^N-labeled Pmt3, revealing the interaction between Slx1^RING+CT^-Slx4^CCD^ and Pmt3 (Fig. 4A). Neither Slx1^RING^-Slx4^CCD^ nor Slx1^RING+CT SIMmut^-Slx4^CCD^ can bind Pmt3 (Fig. 4B). Moreover, ITC showed that Slx1 (Slx1^RING+CT^, residues: 176-271)-Slx4^CCD^ complex has no interaction with ubiquitin (data not shown). Therefore, we conclude that the C-terminal tail of Slx1 has the specificity for the SUMO binding.

## Discussion

In this study, we deciphered the structural mechanism of *S. pombe* Slx1-Slx4 complex formation by determining the crystal structure of the Slx1 C-terminal zinc finger domain in complex with Slx4 C-terminal four-helix bundle domain. Additionally, we demonstrated the C-terminal tail of *S. pombe* Slx1 contains a SUMO-interacting motif and can bind *S. pombe* SUMO, Pmt3.

Slx1 C-terminal domain was originally classified into a PHD finger based on the primary sequence information, but the structural information we provided here indicated it possesses structural characteristics of C4HC3-type RING fingers. However, the third and fourth conserved cysteines in Slx1^RING^ are separated by four residues, not one residue in typical C4HC3-type RING fingers (Supplementary Fig. S3). Thus, Slx1^RING^ may be recognized as an atypical C4HC3-type RING finger. This RING finger subgroup includes Slx1 and FANCL (Supplementary Fig. S3).

RING fingers and RING-like domains constitute the majority of ubiquitin E3 ligases, some SP-RING fingers are also found to be SUMO E3 ligases (e.g. Siz1, PIAS1, and Nse2)^25^,^26^,^27^. Human FANCL RING finger is a monomeric ubiquitin E3 ligase that prefers to bind E2-conjucating enzyme Ube2T^22^. Structural superposition of Slx1^RING^-Slx4^CCD^ complex with human FANCL-Ube2T complex revealed the Slx1-Slx4 binding interface is far from the interface with the possible E2 if Slx1 C-terminal RING finger could function as SUMO or ubiquitin E3 ligase (Supplementary Fig. S4). Our *in vitro* SUMOylation assays did not detect Slx1^RING^-Slx4^CCD^ complex could promote SUMOylation on possible substrates such as *S. pombe* Saw1 and Rad22^28^,^29^,^30^ (data not shown). Future cell-based studies are required to explore this possibility. Recent studies showed that human and mouse SLX4 can bind SUMO2/3 chains through the SIM cluster^12^,^13^,^14^, and human SLX1-SLX4 complex function as a SUMO E3 ligase towards itself and its binding protein XPF^13^. It remains unclear whether human SLX1 possesses a SUMO E3 ligase activity. Nonetheless, Slx1 C-terminal RING finger is critical for Slx1-Slx4 complex formation, and the interaction is essential for the endonuclease activity of Slx1. Ubiquitination and SUMOylation are intersected by SUMO-targeted ubiquitin ligases that have both SIMs and RING fingers, such as *S. cerevisiae* Slx5-Slx8 complex and human RNF4^31^. SIMs are responsible for binding SUMOylated proteins, and the RING domain is able to ubiquitinate SUMOylated targets. *S. pombe* Slx1 possesses similar SIM and RING finger domain arrangements with SUMO-targeted ubiquitin ligase, whereas it is unclear whether Slx1 has such a function.

To maintain the genome integrity, DNA damage response (DDR) pathways are developed to sense, transduce, and repair damaged DNA. The DDR process is regulated by post-translational modifications such as phosphorylation, acetylation, ubiquitination, and SUMOylation. A number of proteins involved in DDR signaling including PARP-1, BRCA1, PCNA were found to be SUMOylated, which is critical for modulating their functions. For example, defective SUMOylation impaired the recruitment of BRCA1 and 53BP1 to double strand break sites^32^,^33^. SUMOylation of yeast PCNA recruits Srs2, which have a PCNA interacting motif and SIM, to disrupt Rad51 nucleoprotein filaments^34^,^35^. Human Slx4 can bind several SUMOylated targets (e.g. RPA70), and is recruited to DNA damage sites to facilitate the repair process^12^. A recent study demonstrated that *S. cerevisiae* SUMOylated Saw1, a scaffold protein, can recruit Slx1-Slx4 complex via Slx1-SUMO and Saw1-Slx4 interactions^36^. Subsequently, Saw1 recruits Rad1-Rad10 to cooperate with Slx1-Slx4 to execute cleavage of the DNA lesion in UV excision repair pathway. However, it is unclear which region of *S. cerevisiae* Slx1 interacts with SUMO. In this study, we found the C-terminal tail of *S. pombe* Slx1 can recognize SUMO. It is implied that *S. pombe* Slx1-Slx4 complex may be recruited by SUMOylated proteins to take part in the DNA damage response pathways.

## Methods

### Protein expression and purification

The DNA sequences of *S. pombe* RING finger domain of Slx1 (Slx1^RING^, residues: 176-247) and C-terminal conserved domain of Slx4 (Slx4^CCD^, residues: 352-419) were amplified from the genomic DNA of *S. pombe* by PCR, and cloned into a pETDuet-1-derived vector (Novagen). This construct encodes Slx1^RING^ and an N-terminal hexahistidine (6×His)-tagged Slx4^CCD^ with thrombin cleavage site. Slx1^RING^-Slx4^CCD^ complex were co-expressed in *E. coli* Rosetta (DE3) strain (Novagen) in LB medium (Affymetrix, USA). The bacterial cells were grown at 37 °C to OD_600 nm_~ 0.8 and then induced with 0.3 mM isopropyl-β-D-thiogalactoside (IPTG) for 16–20 hrs at 16 °C. Cells were collected by centrifugation and lysed by sonication in a buffer containing 20 mM Tris-HCl, pH 7.0, 250 mM NaCl, 2 mM phenylmethylsulfonyl fluoride (PMSF), 0.2 mg/ml lysosome, and 1.5% glycerol. The protein complex was purified by HisTrap^TM^ affinity column followed by thrombin cleavage of the 6×His Tag overnight at 18 °C, gel filtration Superdex^TM^ 75 column and anion-exchange Resource Q column. The protein complex was concentrated to 15 mg/ml in 20 mM Tris-HCl, pH 7.0, 100 mM NaCl, 1mM tris-(2-carboxyethyl)phosphine (TCEP). The purity of the protein complex was analyzed by sodium dodecyl sulfate polyacrylamide gel electrophoresis (SDS-PAGE). The preparation of the C-terminal domain of Slx1^RING+CT^ (residues: 176–271) or Slx1^RING+CT SIMmut^ (IIDLE is mutated to AADAE) complexed with Slx4^CCD^ was conducted with a similar procedure. Slx1^RING+CT SIMmut^ was generated by PCR-driven overlap extension with the wild-type plasmid as the template. The coding DNA sequence of mature Pmt3 (residues: 1–111) was amplified by PCR from the *S. pombe* cDNA, subsequently cloned into a pET28a vector. The expression of mature Pmt3 was carried out with a similar procedure as described above. Uniformly ^15^N-labeled Pmt3 was prepared by growing bacteria in M9 medium with ^15^NH_4_Cl as the sole nitrogen source. The purification of Pmt3 was conducted with a similar procedure as described above.

### Crystallization and structure determination

Crystals were obtained at 18 °C using sitting drop vapor-diffusion method by mixing 1 μl protein complex sample with 1 μl reservoir solution (1.3 M ammonium sulfate, 0.1 M Bis-Tris, pH 6.8). For data collection, the crystal was soaked in the cryoprotectant (reservoir solution supplemented with 25% v/v glycerol), and flash-frozen in the liquid nitrogen. The anomalous diffraction data were collected at the zinc peak wavelength (1.2824 Å) at 100K at the Shanghai Synchrotron Radiation Facility (SSRF). The data were indexed, integrated, and scaled with the program HKL2000^37^. Based on the highest resolution bin *R*_merge_<50%, the resolution cut-off was defined to 2.0 Å. The structure was determined by zinc single-wavelength anomalous dispersion (SAD) using the PHENIX AutoSol program^38^. The initial model was refined using the programs Refmac5^39^ and rebuilt interactively using the program Coot^40^. The final model was evaluated with the program MolProbity^41^. Crystallographic parameters of the structure are listed in Table 1. All structure figures were prepared with PyMOL^42^.

### Isothermal titration calorimetry

Calorimetric experiments were carried out at 25 °C with a MicroCal ITC200 instrument (GE Healthcare Life Sciences, UK). The protein samples were prepared in an ITC buffer consisted of 20 mM sodium phosphate, pH 6.8, 100 mM NaCl. 120 μM Slx1^RING+CT^-Slx4^CCD^, Slx1^RING^-Slx4^CCD^, or Slx1^RING+CT SIMmut^-Slx4^CCD^ sample was transferred into the sample cell. Experiments were initiated upon addition of 2.37 mM Pmt3 in a manner of 39 serial injections of 1 μl each. The first titration was 0.5 μl, and was removed in the following data analysis. Control ITC titrations of Pmt3 into the buffer were conducted with the same procedure. The delay time between the injections was 150 s. The net heat release of each injection was calculated by subtracting the control heat absorption. Data were analyzed using MicroCal Origin software. The experiments were performed three times.

### NMR titration

All NMR spectra were collected at 298 K on a Bruker Avance 600 MHz NMR spectrometer with cryoprobe. NMR samples were prepared in a buffer containing 20 mM sodium phosphate, pH 6.8, 100 mM NaCl, and 10% D_2_O (vol/vol). Titrations were performed by recording a series of 2D ^15^N HSQC spectra on uniformly ^15^N-labeled Pmt3 (0.27 mM) in the presence of different concentrations of Slx1^RING+CT^-Slx4^CCD^, Slx1^RING^-Slx4^CCD^, or Slx1^RING+CT SIMmut^-Slx4^CCD^ ranging from 0 to 0.81 mM. NMR data were processed by NMRPipe^43^ and subsequently analyzed by NMRView^44^.

## Additional Information

**Accession codes:** The final atom coordinates and structure factors for Slx1^RING^-Slx4^CCD^ have been deposited in the Protein Data Bank (http://www.pdb.org/) with accession code 4ZDT.

**How to cite this article**: Lian, F. M. *et al.* Crystal structure and SUMO binding of Slx1-Slx4 complex. *Sci. Rep.* **6**, 19331; doi: 10.1038/srep19331 (2016).

## Supplementary Material

Supplementary Information

## Figures and Tables

**Table 1:** Data collection and refinement statistics.

	Slx1^RING^ Slx4^CCD^
**Data collection**	
Space group	*P4*_*1*_
Cell dimensions	
a, b, c (Å)	85.23, 85.23, 74.84
α, β, γ (º)	90.00, 90.00, 90.00
Wavelength (Å)	1.2824
Resolution (Å)^a^	50.00-2.00 (2.07-2.00)
Unique reflections	36121 (3574)
*R*_merge_^b^ (%)	10.9 (48.3)
*I / σI*	28.9 (7.8)
CC1/2 (%)	99.6 (97.0)
Completeness (%)	100.0 (100.0)
Redundancy	14.9
**Refinement**	
Resolution (Å)	38.10-2.00
No. reflections	34383
*R*_work_^c^/*R*_free_^d^ (%)	21.51/23.63
No. atoms	
Protein	2217
Zinc/Sulfate/Glycerol	4/5/30
Water	107
Mean B factors (Å^2^)	
Protein	32.1
Zinc/Sulfate/Glycerol	38.7/32.8/52.1
Water	32.2
R.m.s deviations^e^	
Bond lengths (Å)	0.012
Bond angles (º)	1.139
Ramachandran plot^f^ (residues, %)	
Most favored (%)	98.1
Additional allowed (%)	1.9
Outliers (%)	0

^a^The values in parentheses refer to statistics in the highest bin.

^b^*R*_merge_=∑_hkl_∑_i_|I_i_(hkl)− <I(hkl)>| / ∑_hkl_∑_i_I_i_(hkl).

^c^*R*_work_=∑_h_|Fo(h)−Fc(h)|/∑_h_Fo(h).

^d^*R*_free_ was calculated with 5% of the data excluded from the refinement.

^e^Root-mean square-deviation from ideal values^45^.

^f^Categories were defined by MolProbity^41^.

## References

[b1] Svendsen J. M. *et al.* Mammalian BTBD12/SLX4 assembles a Holliday junction resolvase and is required for DNA repair. Cell 138, 63–77 (2009). 19596235 10.1016/j.cell.2009.06.030 PMC2720686

[b2] Fekairi S. *et al.* Human SLX4 is a Holliday junction resolvase subunit that binds multiple DNA repair/recombination endonucleases. Cell 138, 78–89 (2009). 19596236 10.1016/j.cell.2009.06.029 PMC2861413

[b3] Munoz I. M. *et al.* Coordination of structure-specific nucleases by human SLX4/BTBD12 is required for DNA repair. Mol. Cell 35, 116–127 (2009). 19595721 10.1016/j.molcel.2009.06.020

[b4] Kim Y. *et al.* Regulation of multiple DNA repair pathways by the Fanconi anemia protein SLX4. Blood 121, 54–63 (2013). 23093618 10.1182/blood-2012-07-441212 PMC3538331

[b5] Castor D. *et al.* Cooperative control of holliday junction resolution and DNA repair by the SLX1 and MUS81-EME1 nucleases. Mol. Cell 52, 221–233 (2013). 24076219 10.1016/j.molcel.2013.08.036 PMC3808987

[b6] Wyatt H. D., Sarbajna S., Matos J. & West S. C. Coordinated actions of SLX1-SLX4 and MUS81-EME1 for Holliday junction resolution in human cells. Mol. Cell 52, 234–247 (2013). 24076221 10.1016/j.molcel.2013.08.035

[b7] Klein Douwel D. *et al.* XPF-ERCC1 acts in Unhooking DNA interstrand crosslinks in cooperation with FANCD2 and FANCP/SLX4. Mol. Cell 54, 460–471 (2014). 24726325 10.1016/j.molcel.2014.03.015 PMC5067070

[b8] Hodskinson M. R. *et al.* Mouse SLX4 is a tumor suppressor that stimulates the activity of the nuclease XPF-ERCC1 in DNA crosslink repair. Mol. Cell 54, 472–484 (2014). 24726326 10.1016/j.molcel.2014.03.014 PMC4017094

[b9] Wan B. *et al.* SLX4 assembles a telomere maintenance toolkit by bridging multiple endonucleases with telomeres. Cell Rep. 4, 861–869 (2013). 24012755 10.1016/j.celrep.2013.08.017 PMC4334113

[b10] Laguette N. *et al.* Premature activation of the SLX4 complex by Vpr promotes G2/M arrest and escape from innate immune sensing. Cell 156, 134–145 (2014). 24412650 10.1016/j.cell.2013.12.011

[b11] Kim Y. *et al.* Mutations of the SLX4 gene in Fanconi anemia. Nat. Genet. 43, 142–146 (2011). 21240275 10.1038/ng.750 PMC3345287

[b12] Ouyang J. *et al.* Noncovalent interactions with SUMO and ubiquitin orchestrate distinct functions of the SLX4 complex in genome maintenance. Mol. Cell 57, 108–122 (2015). 25533185 10.1016/j.molcel.2014.11.015 PMC4289429

[b13] Guervilly J. H. *et al.* The SLX4 complex is a SUMO E3 ligase that impacts on replication stress outcome and genome stability. Mol. Cell 57, 123–137 (2015). 25533188 10.1016/j.molcel.2014.11.014

[b14] Gonzalez-Prieto R., Cuijpers S. A., Luijsterburg M. S., van Attikum H. & Vertegaal A. C. SUMOylation and PARylation cooperate to recruit and stabilize SLX4 at DNA damage sites. EMBO Rep. 16, 512–519 (2015). 25722289 10.15252/embr.201440017 PMC4388617

[b15] Stoepker C. *et al.* SLX4, a coordinator of structure-specific endonucleases, is mutated in a new Fanconi anemia subtype. Nat. Genet. 43, 138–141 (2011). 21240277 10.1038/ng.751

[b16] Mullen J. R., Kaliraman V., Ibrahim S. S. & Brill S. J. Requirement for three novel protein complexes in the absence of the Sgs1 DNA helicase in Saccharomyces cerevisiae. Genetics 157, 103–118 (2001). 11139495 10.1093/genetics/157.1.103 PMC1461486

[b17] Fricke W. M. & Brill S. J. Slx1-Slx4 is a second structure-specific endonuclease functionally redundant with Sgs1-Top3. Genes Dev. 17, 1768–1778 (2003). 12832395 10.1101/gad.1105203 PMC196184

[b18] Kaliraman V. & Brill S. J. Role of SGS1 and SLX4 in maintaining rDNA structure in Saccharomyces cerevisiae. Curr. Genet. 41, 389–400 (2002). 12228808 10.1007/s00294-002-0319-6 PMC2804045

[b19] Coulon S. *et al.* Slx1-Slx4 are subunits of a structure-specific endonuclease that maintains ribosomal DNA in fission yeast. Mol. Biol. Cell 15, 71–80 (2004). 14528010 10.1091/mbc.E03-08-0586 PMC307528

[b20] Gaur V. *et al.* Structural and Mechanistic Analysis of the Slx1-Slx4 Endonuclease. Cell Rep. 10, 1467–1476 (2015). 10.1016/j.celrep.2015.02.019 PMC4407285 25753413

[b21] Holm L. & Rosenstrom P. Dali server: conservation mapping in 3D. Nucleic Acids Res. 38, W545–549 (2010). 20457744 10.1093/nar/gkq366 PMC2896194

[b22] Hodson C., Purkiss A., Miles J. A. & Walden H. Structure of the human FANCL RING-Ube2T complex reveals determinants of cognate E3-E2 selection. Structure 22, 337–344 (2014). 24389026 10.1016/j.str.2013.12.004 PMC3979106

[b23] Plechanovova A., Jaffray E. G., Tatham M. H., Naismith J. H. & Hay R. T. Structure of a RING E3 ligase and ubiquitin-loaded E2 primed for catalysis. Nature 489, 115–120 (2012). 22842904 10.1038/nature11376 PMC3442243

[b24] Bentley M. L. *et al.* Recognition of UbcH5c and the nucleosome by the Bmi1/Ring1b ubiquitin ligase complex. EMBO J. 30, 3285–3297 (2011). 21772249 10.1038/emboj.2011.243 PMC3160663

[b25] Takahashi Y., Kahyo T., Toh E. A., Yasuda H. & Kikuchi Y. Yeast Ull1/Siz1 is a novel SUMO1/Smt3 ligase for septin components and functions as an adaptor between conjugating enzyme and substrates. J. Biol. Chem. 276, 48973–48977 (2001). 11577116 10.1074/jbc.M109295200

[b26] Kahyo T., Nishida T. & Yasuda H. Involvement of PIAS1 in the sumoylation of tumor suppressor p53. Mol. Cell 8, 713–718 (2001). 11583632 10.1016/s1097-2765(01)00349-5

[b27] Potts P. R. & Yu H. Human MMS21/NSE2 is a SUMO ligase required for DNA repair. Mol. Cell. Biol. 25, 7021–7032 (2005). 16055714 10.1128/MCB.25.16.7021-7032.2005 PMC1190242

[b28] Psakhye I. & Jentsch S. Protein group modification and synergy in the SUMO pathway as exemplified in DNA repair. Cell 151, 807–820 (2012). 23122649 10.1016/j.cell.2012.10.021

[b29] Cremona C. A. *et al.* Extensive DNA damage-induced sumoylation contributes to replication and repair and acts in addition to the mec1 checkpoint. Mol. Cell 45, 422–432 (2012). 22285753 10.1016/j.molcel.2011.11.028 PMC3340930

[b30] Ho J. C., Warr N. J., Shimizu H. & Watts F. Z. SUMO modification of Rad22, the Schizosaccharomyces pombe homologue of the recombination protein Rad52. Nucleic Acids Res. 29, 4179–4186 (2001). 11600706 10.1093/nar/29.20.4179 PMC60211

[b31] Prudden J. *et al.* SUMO-targeted ubiquitin ligases in genome stability. EMBO J. 26, 4089–4101 (2007). 17762865 10.1038/sj.emboj.7601838 PMC2230673

[b32] Morris J. R. *et al.* The SUMO modification pathway is involved in the BRCA1 response to genotoxic stress. Nature 462, 886–890 (2009). 20016594 10.1038/nature08593

[b33] Galanty Y. *et al.* Mammalian SUMO E3-ligases PIAS1 and PIAS4 promote responses to DNA double-strand breaks. Nature 462, 935–939 (2009). 20016603 10.1038/nature08657 PMC2904806

[b34] Krejci L. *et al.* DNA helicase Srs2 disrupts the Rad51 presynaptic filament. Nature 423, 305–309 (2003). 12748644 10.1038/nature01577

[b35] Armstrong A. A., Mohideen F. & Lima C. D. Recognition of SUMO-modified PCNA requires tandem receptor motifs in Srs2. Nature 483, 59–63 (2012). 22382979 10.1038/nature10883 PMC3306252

[b36] Sarangi P. *et al.* A versatile scaffold contributes to damage survival via sumoylation and nuclease interactions. Cell Rep. 9, 143–152 (2014). 25263559 10.1016/j.celrep.2014.08.054 PMC4280569

[b37] Otwinowski Z. & Minor W. Processing of X-ray diffraction data collected in oscillation mode. Methods Enzymol. 276, 307–326 (1997). 10.1016/S0076-6879(97)76066-X 27754618

[b38] Terwilliger T. C. *et al.* Decision-making in structure solution using Bayesian estimates of map quality: the PHENIX AutoSol wizard. Acta Crystallogr. D Biol. Crystallogr. 65, 582–601 (2009). 19465773 10.1107/S0907444909012098 PMC2685735

[b39] Murshudov G. N., Vagin A. A. & Dodson E. J. Refinement of macromolecular structures by the maximum-likelihood method. Acta Crystallogr. D Biol. Crystallogr. 53, 240–255 (1997). 15299926 10.1107/S0907444996012255

[b40] Emsley P. & Cowtan K. Coot: model-building tools for molecular graphics. Acta Crystallogr. D Biol. Crystallogr. 60, 2126–2132 (2004). 15572765 10.1107/S0907444904019158

[b41] Chen V. B. *et al.* MolProbity: all-atom structure validation for macromolecular crystallography. Acta Crystallogr. D Biol. Crystallogr. 66, 12–21 (2010). 20057044 10.1107/S0907444909042073 PMC2803126

[b42] DeLano W. The PyMOL Molecular Graphics System. (2002).

[b43] Delaglio F. *et al.* NMRPipe: a multidimensional spectral processing system based on UNIX pipes. J. Biomol. NMR 6, 277–293 (1995). 8520220 10.1007/BF00197809

[b44] Johnson B. A. Using NMRView to visualize and analyze the NMR spectra of macromolecules. Methods Mol. Biol. 278, 313–352 (2004). 15318002 10.1385/1-59259-809-9:313

[b45] Engh R. A. & Huber R. Accurate bond and angle parameters for X-ray protein structure refinement. Acta Crystallogr. A Found. Adv. 47, 392–400 (1991).

