# Supplementary material for: Crystal structure and SUMO binding of Slx1-Slx4 complex
Source: Sci Rep. 2016 Jan 20;6:19331. doi: 10.1038/srep19331 (PMC4726241; doi:10.1038/srep19331)
Supplement: Supplementary Information [file srep19331-s1.pdf]

Crystal structure and SUMO binding of Slx1-Slx4 complex

Fu-Ming Lian, Si Xie, Chengmin Qian*

School of Biomedical Sciences, The University of Hong Kong, Hong Kong, China

*Correspondence should be addressed to C.Q. (cmqian@hku.hk).

Supplementary figures

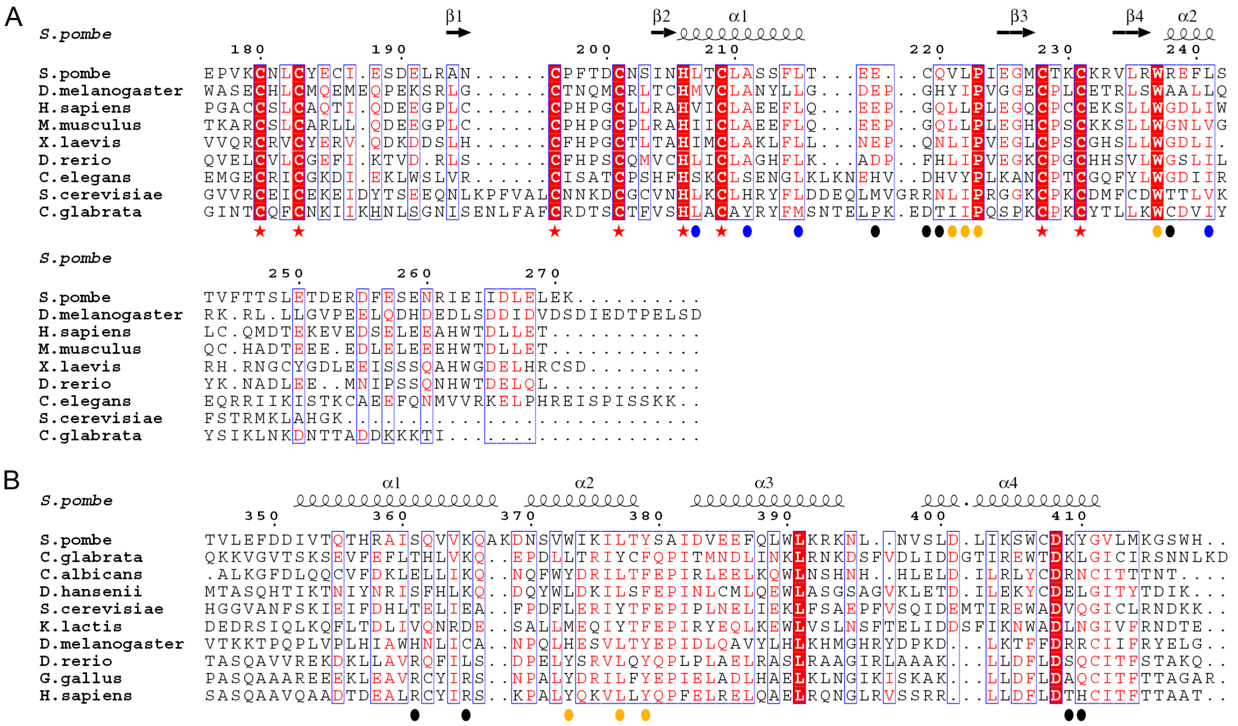

Supplementary Figure S1. Multiple-sequence alignments of Slx1 and Slx4. A. Sequence alignment of the C-terminal domain of Slx1 orthologs from different species. **B.** Sequence alignment of the C-terminal domain of Slx4 orthologs from different species. The secondary structural elements of *S. pombe* Slx1 and Slx4 are shown above the sequences. The conserved cysteine/histidine residues coordinated zinc ions are marked with red stars. The residues contributed to complex interface are marked with blue (hydrophobic interactions), black (hydrogen bonds), and orange (both hydrophobic interactions and hydrogen bonds) cycles.

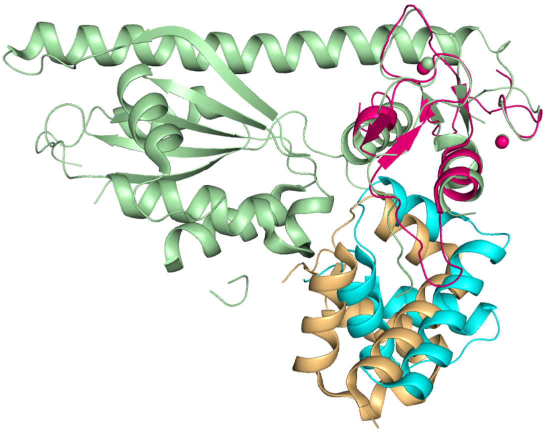

Supplementary Figure S2. Superposition of *S. pombe* Slx1^{RING}-Slx4^{CCD} with *Candida glabrata* Slx1-Slx4^{CCD}. *S. pombe* Slx1^{RING}, Slx4^{CCD}, *C. glabrata* Slx1, and Slx4^{CCD} are colored in pink, cyan, green, and orange, respectively. Pink and green spheres indicate zinc ions.

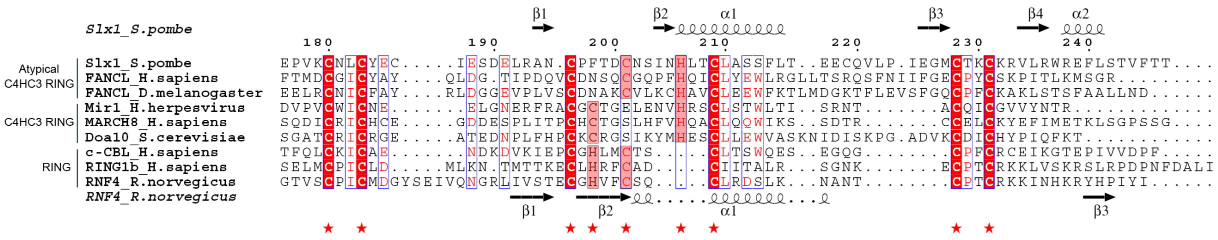

Supplementary Figure S3. Structure-based sequence alignment of three types of RING fingers. The multi-sequence alignment was conducted using programs Clustral W[1] and ESPript[2]. The conserved secondary structural elements of *S. pombe* Slx1 and *R. norvegicus* RNF4 are shown above and below the sequences. The conserved cysteine/histidine residues coordinated zinc ions are marked with red stars. PDB codes used for alignment are as follows: 4CCG (*Homo sapiens* FANCL), 3K1L (*Drosophila melanogaster* FANCL)[3], 1VYX (Human herpesvirus 8 Mir1)[4], 2D8S (*Homo sapiens* MARCH8), 2M6M (*Saccharomyces cerevisiae* Doa10), 1FBV (*Homo sapiens* c-CBL)[5], 3RPG (*Homo sapiens* RING-1b), and 4AP4 (*Rattus norvegicus* RNF4).

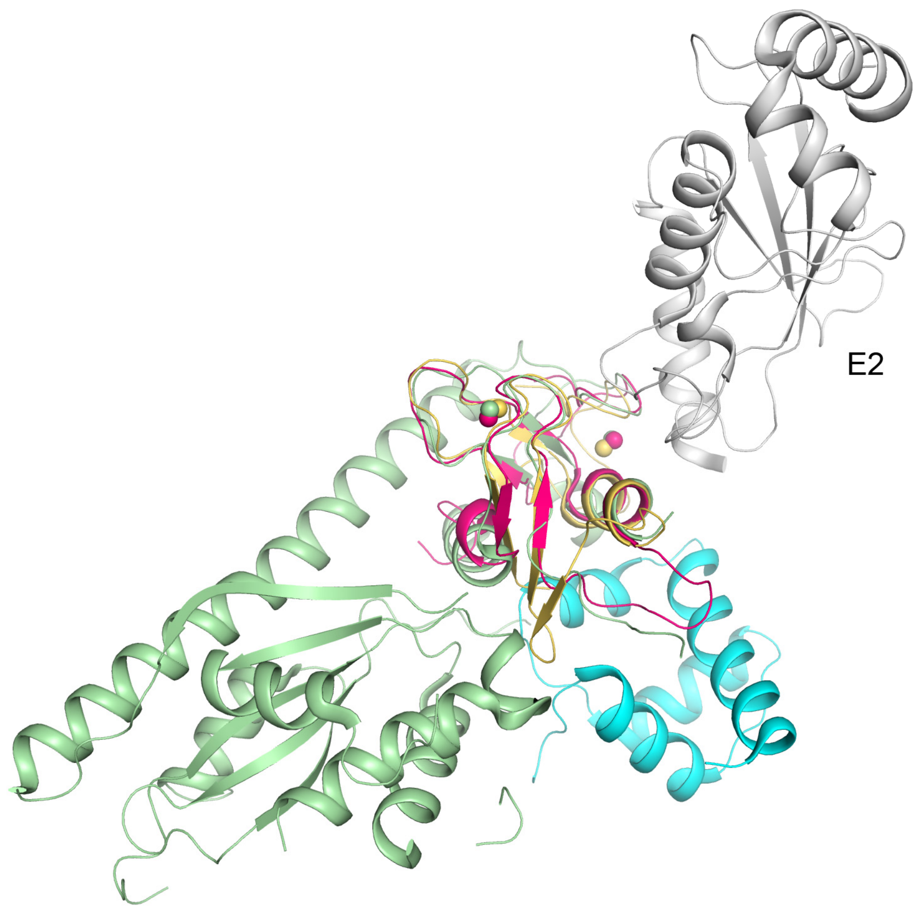

Supplementary Figure S4. Superposition of *S. pombe* Slx1^{RING}-Slx4^{CCD} with *Candida glabrata* Slx1, and *Homo sapiens* FANCL-Ube2T. *S. pombe* Slx1^{RING}, Slx4^{CCD}, *C. glabrata* Slx1, FANCL, and Ube2T are colored in pink, cyan, green, yellow, and gray, respectively. Spheres indicate zinc ions.

References

1. Larkin, M.A. *et al.* Clustal W and Clustal X version 2.0. *Bioinformatics* **23,** 2947-2948 (2007).
2. Robert, X. & Gouet, P. Deciphering key features in protein structures with the new ENDscript server. *Nucleic Acids Res.* **42,** W320-324 (2014).
3. Cole, A.R., Lewis, L.P. & Walden, H. The structure of the catalytic subunit FANCL of the Fanconi anemia core complex. *Nat. Struct. Mol. Biol.* **17,** 294-298 (2010).
4. Dodd, R.B. *et al.* Solution structure of the Kaposi's sarcoma-associated herpesvirus K3 N-terminal domain reveals a Novel E2-binding C4HC3-type RING domain. *J. Biol. Chem.* **279,** 53840-53847 (2004).
5. Zheng, N., Wang, P., Jeffrey, P.D. & Pavletich, N.P. Structure of a c-Cbl-UbcH7 complex: RING domain function in ubiquitin-protein ligases. *Cell* **102,** 533-539 (2000).
